# Tolerance to Copper and to Salinity in *Daphnia longispina*: Implications within a Climate Change Scenario

**DOI:** 10.1371/journal.pone.0068702

**Published:** 2013-08-26

**Authors:** João Leitão, Rui Ribeiro, Amadeu M. V. M. Soares, Isabel Lopes

**Affiliations:** 1 Department of Biology, University of Aveiro, Aveiro, Portugal; 2 IMAR–Instituto do Mar, Department of Life Sciences, University of Coimbra, Coimbra, Portugal; 3 Department of Biology and CESAM, University of Aveiro, Aveiro, Portugal; Helmholtz Centre for Environmental Research – UFZ, Germany

## Abstract

Considering IPPC climate change scenarios, it is pertinent to predict situations where coastal ecosystems already impacted with chemical contamination became exposed to an additional stressor under a future scenario of seawater intrusion. Accordingly, the present study aimed at evaluating if a negative association between tolerance to a metal and to saltwater exists among genotypes of a freshwater organism. For this, five clonal lineages of the cladoceran *Daphnia longispina* O.F. Müller, exhibiting a differential tolerance to lethal levels of copper, were selected. Each clonal lineage was exposed to lethal and sublethal concentrations of sodium chloride (assumed as a protective surrogate to evaluate the toxicity of increased salinity to freshwater organisms). Mortality, time to release the first brood and total number of neonates per female were monitored and the somatic growth rate and intrinsic rate of natural increase were computed for each clonal lineage. Data here obtained were compared with their lethal responses to copper and significant negative correlations were found. These results suggest that genetically eroded populations of *D. longispina*, due to copper or salinity, may be particularly susceptible to a later exposure to the other contaminant supporting the multiple stressors differential tolerance.

## Introduction

At present, the rising of sea level is recognized as one of the major issues in environmental protection, especially when considering the conservation of biodiversity of low-lying coastal freshwater ecosystems [1]. Accordingly, several works have been carried out to determine the sensitivity of freshwater species, inhabiting these ecosystems, to increased salinity and to understand what ecological changes may occur due to seawater intrusion (e.g. [2]–[5]). Most of these works were focused on understanding the intrinsic sensitivity of tested organisms to increased salinity, induced by artificial seawater or by the use of one salt (usually NaCl, which is recognized as representing a worst case scenario; [6]), under optimal conditions (e.g. [3], [7], [8]). However, it is pertinent to consider scenarios where coastal ecosystems are already impacted with chemical contamination, and that the biota inhabiting these ecosystems will be exposed to an additional stressor under a scenario of future seawater intrusion. It has been shown that if populations of freshwater organisms are exposed to chemical contamination, then its genetic erosion may occur through the elimination of the most sensitive genotypes (e.g. [9]–[15]). Whether the remaining tolerant genotypes are also tolerant to other type of contamination will determine the persistence of the already genetically eroded population under a situation of future contaminants' inputs. If variability in tolerance exists within populations, two alternative scenarios may occur. First, if a positive association between tolerance to the different chemicals exists, then the genotypes surviving the input of a first chemical will be able to cope with the ulterior input of another chemical. Second, if negative linkages between tolerance to the two contaminants exist – the multiple stressors differential tolerance hypothesis [14], [15] – then at least some genotypes remaining in the population (tolerant to the first chemical) may disappear after the exposure to the second chemical to which they are sensitive to. Only the tolerant individuals to both chemicals will persist after both exposures. The extreme configuration of this later scenario would constitute the worst case: a negative correlation would lead to an increased risk of population extinction [14], [16].

Positive associations between tolerance to several chemicals has already been reported. As an example, Lopes et al. [10] observed that lethal tolerance to copper was positively associated (*r* = 0.73) with lethal tolerance to zinc in 12 clonal lineages of the cladoceran *Daphnia longispina*. Also, Soldo and Behra [17] carried out experiences with communities of periphyton and found that a long-term exposure to copper also induced an increased resistance to zinc, nickel and silver. However, simultaneous resistance to NaCl and to other chemicals has rarely been addressed and the existing published works were mostly carried out with plant species [18]–[21]. For example, Hodson et al. [18] compared the sensitivity to several ions (lithium, potassium, rubidium, caesium, magnesium, calcium) of a clone of the grass *Agrostis stolonifera* from a salt marsh with another one from an inland ecosystem. They observed that the former was always more tolerant to increased salinity than the inland one. Also, Shah et al. [20] exposed cell lines of the monocotyledon *Oryza sativa*, adapted to LiCl or to NaCl, to several concentrations of these chemicals and observed that adapted lines were considerably more tolerant than the non adapted lines to both chemicals. Nevertheless, independency between tolerance to NaCl and other chemicals have also been reported. As an example, Wu et al. [22] studied the tolerance of thirteen lines of the tall fescue *Festuca arundinacea* to NaCl and selenium and found independency in the tolerance to these two chemicals.

The present work aimed at testing the multiple stressors differential tolerance hypothesis [14]–[15] with the freshwater cladoceran *D. longispina* exposed to copper and to NaCl.

## Materials and Methods

### Test organisms

Five clonal lineages of *Daphnia longispina* O.F. Müller were selected to conduct this work: N91, N116, N31, E99 and E89 (Table 1 shows the allozyme and microsatellite profiles, determined by [23], [24], of these five clonal lineages). These lineages were derived from two field populations: one inhabiting a reference site, and the other inhabiting an acid mine drainage (AMD) historically impacted site; both located at the aquatic system of an abandoned cupric-pyrite mine (São Domingos mine, Southeast Portugal) [25]. This mine was active for almost 100 years and the ore exploitation ended approximately fifty years ago. However, the continuous oxidation of the mine tailings produces an acidic effluent contaminated with metals [25], [26]. Therefore, the sources of contamination of the historically impacted aquatic system are hydrogen ions and metals which are present in the AMD (pH≈2.1, contaminated with Fe, Al, Zn, Cu, Mn, Co, Ni, Cd, Pb, Cr, As, in decreasing order), and no other significant contamination sources are present [25], [26]. The five clonal lineages were maintained in laboratory, for more than 100 generations, under controlled conditions of temperature 19 to 21°C, and photoperiod of 16∶8 h L∶D in American Society for Testing and Materials (ASTM) hardwater [27], with the addition of vitamins (7.5 mg/L B1, 1 mg/L B12, and 0.75 mg/L biotin) and a standard organic extract from the algae *Ascophyllum nodosum* “Marinure 25” (Pann Britannica, Waltham Abbey, UK) [28]. Organisms were fed every day with 3×10^5^ cells/mL/d of the green algae *Pseudokirchneriella subcapitata* (Korshikov) Hindak (formerly known as *Selenastrum capricornutum* Printz) and water medium was renewed every other day. These five clonal lineages were chosen for this study according to their range of genetically determined tolerance to lethal levels of copper, which was previously characterized by Venâncio [29]. During the 15-years long laboratory culture, the tolerance of *D. longispina* genotypes to copper remained unchanged [29]. Furthermore, no differences in tolerance to sublethal levels of copper among the studied genotypes were ever found. Therefore, as sublethal levels of copper could not lead to genetic erosion, this comparison with the tolerance to NaCl was not addressed.

**Table 1:** Allozyme and microsatellite profiles of the five clonal lineages of *Daphnia longispina* (from [23], [24]).

Lineage	Allozyme profile	Microsatellite profile
E89	a	IV
N91	c	I
N31	c	VI
E99	c	I
N116	e	VIII

### Lethal toxicity assays

Lethal toxicity assays were carried out with the five clonal lineages by following the standard protocol of OECD [30]. Neonates (>6 and <24 h old) from the third to the fifth brood were exposed to lethal concentrations of sodium chloride and to control (ASTM hardwater). A stock concentration of 5.2 g/L of NaCl (Sigma-Aldrich, St. Louis, MO, USA) was prepared by dissolving this salt in ASTM hardwater. A gradient of 5 concentrations (4.5, 4.0, 3.4, 3.0, and 2.6 g/L) was achieved by diluting the initial stock concentration with ASTM hardwater. Five individuals were exposed simultaneously per 42-mL glass vessels filled with 20 mL of the test solution, with four replicates per treatment. The assays were performed under the same conditions of temperature and photoperiod as those described for the laboratory cultures, with neither the addition of vitamins, nor the organic extract, nor algae. Organisms were exposed for a period of 48 h and immobilization (here considered as mortality; organisms remaining immobile during 15 s after gentle prodding) being checked at 24 and 48 h. Salinity (HANNA Instruments Seawater Refractometer HI 96822, Woonsocket, RI, USA), conductivity (Wissenschaftlich Technische Werkstätten LF92 conductivity meter, Weilheim, Germany), pH (Wissenschaftlich Technische Werkstätten 537 pH meter), and dissolved oxygen (DO) (Wissenschaftlich Technische Werkstätten OXI92 oxygen meter) were measured for each treatment at the beginning and end of the assay.

### Sublethal toxicity assays

To assess the effects of increased salinity on sublethal responses of the five clonal lineages of *D. longispina*, the standard protocol of OECD [31], was followed up. Ten neonates (>6 and <24 h old), from the third to the fifth brood, of each clonal lineage were exposed individually, under the same controlled conditions of temperature and photoperiod as for the laboratory cultures, to a range of five NaCl concentrations and a control (ASTM hardwater). Test concentrations ranged between 0.72 and 1.5 g/L of NaCl (using a dilution factor of 1.2×) and from 1 to 2.16 g/L in the case of the E89 clonal lineage. These test concentrations were prepared by dissolving NaCl in ASTM hardwater, as described for the lethal toxicity assays. Each individual was introduced in 42-mL glass vessels filled with 20 mL of test solution with the addition of “Marinure 25” and the green algae *P. subcapitata* (3×10^5^ cells/mL/d). Organisms were fed daily, medium being renewed every other day. The assay ended when all the ten individuals in the control released the third brood. The following endpoints were monitored: time to first brood, total number of neonates produced per female, somatic growth rate, and the intrinsic rate of natural increase. Salinity, conductivity, pH, and DO were measured at the new and old medium during renewal.

To compute somatic growth rate, at the beginning of the assay, the body lengths of a subsample of twelve neonates (from the same batch as those used for the toxicity assay) were measured and, at the end of the assay, all females were also measured. The somatic growth rate (d^−1^) was estimated according to the following equation:
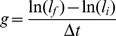
where *g* corresponds to somatic growth rate, *l_i_* and *l_f_* are the initial and final body size (mm) of the daphnids respectively, and Δ*t* corresponds to the time interval.

The intrinsic rate of natural increase (r) was computed for each clonal lineage, at the end of the sublethal toxicity assays, using Lotka's equation:
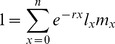
with *l_x_* being the age-specific survivorship; *m_x_* the number of neonates at day *x*; and *x* the age in days. The estimation of the standard errors for *r* was done through the jackknife technique [32].

### Data analysis

Median, lower and upper quartile lethal concentrations of NaCl (LC_50_, LC_25_, and LC_75_, respectively) and the respective 95% confidence limits were calculated for each clonal lineage after 48 hours of exposure, through probit analysis using the software Priprobit [33]. Median, lower and upper quartile sublethal concentrations of NaCl for reproduction (EC_50_, EC_25_, and EC_75_, respectively) were calculated for each clonal lineage with a logistic model, using the software Statistica 8.0 (StatSoft, Tulsa, OK, USA). For each metal, a clonal lineage was categorised as critically sensitive if its LC_75,48 h_ or EC_75_ was close to or below the average of the set of LC_50,48 h_ or EC_50_ for the five clonal lineages, respectively. For each pair of metals, inversely sensitive clonal lineages, of special concern within this study rationale, were those critically sensitive to one of the chemical (NaCl or copper) but not to the other. Therefore, safely co-resistant clonal lineages were those neither critically co-sensitive nor inversely sensitive.

For comparative purposes, the among genotypes variation was quantified with the coefficients of variation (CV) of the median lethal/sublethal concentrations and the within genotypes variation with the relative spread of sensitivity of each clonal lineage: the difference between the lower (LC_25,48 h_/EC_25_) and upper (LC_75,48 h_/EC_75_) quartiles relatively to the median (LC_50,48 h_/EC_50_), in percentage.

To quantify the association between LC_50_ and EC values of NaCl and between the tolerance to copper and to NaCl, Pearson's correlation coefficients were computed. Correlations were calculated using the software Statistica.

To test the significance of the effects of NaCl on sublethal responses, a one-way analysis of variance was carried out, after confirming the normality and homoscedasticity with the Shapiro-Wilk's and Bartlett's tests, respectively. Whenever significant differences were found, a Dunnett's multiple comparison test was applied to determine which concentrations provoked responses significantly different from the control.

## Results

### Lethal assays

During the lethal assay with the five clonal lineages of *D. longispina* exposed to NaCl, the values of pH ranged from 7.7 and 8.4 and the dissolved oxygen was always above 7.4 mg/L. The highest variability observed in conductivity, during the assay, was 0.7 mS/cm.

Median lethal concentrations (LC_50_) after 48 h of exposure to NaCl were similar among the five clonal lineages, differing by less than 1.15 times after 48 h of exposure (Table 2). Average values of LC_50_ were 2.69 g/L, with a coefficient of variation of 6.82%.

**Table 2:** Values of the median lethal concentrations (respective 95% confidence limits) of copper (adapted from [28]) and of median, lower and upper quartile lethal concentrations of NaCl (LC_25,48 h_, LC_50,48 h_, LC_75,48 h_, respectively) for the five clonal lineages of *Daphnia longispina*, after being exposed to this metal for 48 h.

	LC_50,48h_ Cu	LC_25,48h_ NaCl	LC_50,48h_ NaCl	LC_75,48h_ NaCl
E99	163 (156–170)	2.48 (2.43–2.53)	2.50 (2.47–2.57)	2.57 (2.53–2.62)
N91	158 (138–175)	2.64 (2.47–2.74)	2.80 (2.69–2.92)	2.99 (2.88–3.16)
N116	141 (133–148)	2.57 (2.27–2.74)	2.85 (2.65–3.00)	3.16 (3.01–3.41)
N31	51.6 (49.7–53.5)	2.27 (1.45–2.49)	2.48 (1.90–2.70)	2.72 (2.45–2.91)
E89	18.2 (13.1–21.1)	2.55 (2.24–2.72)	2.82 (2.62–2.97)	3.13 (2.97–3.38)

### Sublethal assays

During the sublethal assays, only slight changes were registered in the physico-chemical parameters. The pH values ranged from 8.0 to 8.3 and dissolved oxygen values were always above 6.9 mg/L. The highest variation registered in conductivity was 0.11 mS/cm.

**Figure 1:**
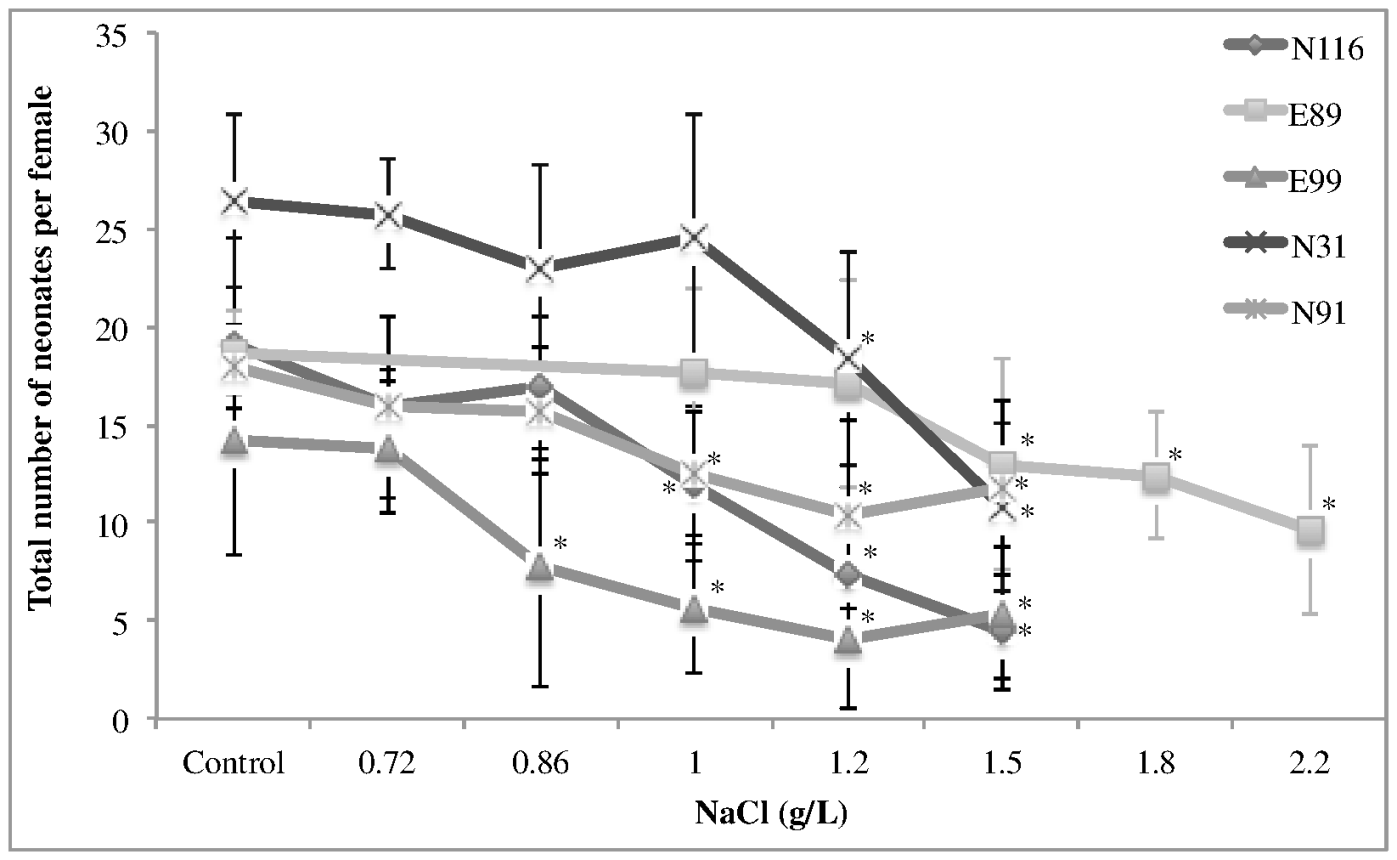
Average of total number of neonates released per female (B) with the respective standard deviation (error bars), of five clonal lineages of *Daphnia longispina* exposed to a gradient of NaCl. *symbolizes significant differences relatively to the control (p≤10^−4^).

Depending on the parameter that was monitored, the five studied clonal lineages of *D. longispina* exhibited different ranks in resistance to NaCl. Considering the time to release the first brood, E89 (the most sensitive to lethal levels of copper) was the most tolerant clonal lineage, as exposure to NaCl induced no change in this parameter (control: 9.3±0.8 days and 2.2 g/L NaCl: 10±1.0 days; F_5,52_ = 1.75, p = 0.14), contrary to all other clonal lineages (time to release the first brood increased 1 to 2 days at the highest NaCl concentrations; F_5,43_≥3.42, p≤10^−6^). Regarding the total number of neonates, the clonal lineage E89 was also the most resistant to NaCl. Only females exposed to concentrations of 1.5 g/L or above released less neonates than the controls, while for all other clonal lineages concentrations below 1.5 g/L induced a significant reduction (Fig. 1; F_5,46_≥6.39, p≤10^−5^; Dunnett's tests: p≤10^−3^). Furthermore, the clonal lineage E89 exhibited the highest values of EC_50_ (2.15 g/L) for reproduction (Table 3). The EC_50_ values for reproduction were 1.3 to 2.5-fold lower than the respective LC_50,48 h_ values.

**Table 3:** Values of NaCl concentrations provoking 25, 50, and 75% (EC_25_, EC_50_, and EC_75_, respectively) decrease in reproduction of the five clonal lineages of *Daphnia longispina*. (respectively 95% confidence limits).

	EC_25_ NaCl	EC_50_ NaCl	EC_75_ NaCl	EC_25_ Curve fit	EC_50_ Curve fit	EC_75_ Curve fit
E99	0.70	0.99	1.41	Rep = 15(±1.5)/(1+[(0.33)×(Conc/0.70)^3.3(±0.1)^]	Rep = 15(±1.5)/(1+(Conc/0.99)^3.3(±0.1)^)	Rep = 15(±1.5)/(1+[(3)×(Conc/1.41)^3.3(±0.1)^]
	(0.45–0.95)	(0.79–1.20)	(1.04–1.79)	R = 0.63	R = 0.63	R = 0.63
N91	0.98	1.73	3.05	Rep = 18(±1.1)/(1+[(0.33)×(Conc/0.98)^2.0(±0.7)^]	Rep = 18(±1.1)/(1+(Conc/1.73)^2.0(±0.7)^)	Rep = 18(±1.1)/(1+[(3)×(Conc/3.05)^2.0(±0.7)^]
	(0.66–1.30)	(1.17–2.29)	(1.05–5.05)	R = 0.58	R = 0.63	R = 0.58
N116	0.92	1.13	1.42	Rep = 19(±1.3)/(1+[(0.33)×(Conc/0.92)^5.1(±1.2)^]	Rep = 19(±1.3)/(1+(Conc/1.13)^5.1(±1.2)^)	Rep = 19(±1.3)/(1+[(3)×(Conc/1.42)^5.1(±1.2)^]
	(0.76–1.07)	(1.01–1.26)	(1.22–1.61)	R = 0.78	R = 0.78	R = 0.78
N31	1.12	1.41	1.70	Rep = 26(±1.4)/(1+[(0.33)×(Conc/1.12)^5.3(±1.4)^]	Rep = 26(±1.4)/(1+(Conc/1.41)^5.3(±1.4)^)	Rep = 26(±1.4)/(1+[(3)×(Conc/1.70)^5.3(±1.4)^]
	(0.96–1.28)	(0.96–1.27)	(1.45–1.94)	R = 0.73	R = 0.73	R = 0.73
E89	1.51	2.15	3.07	Rep = 19(±1.3)/(1+[(0.33)×(Conc/1.51)^3.3(±1.0)^]	Rep = 19(±1.3)/(1+(Conc/2.15)^3.3(±1.0)^)	Rep = 19(±1.3)/(1+[(3)×(Conc/3.07)^3.3(±1.0)^]
	(1.13–1.89)	(1.77–2.53)	(2.01–4.12)	R = 0.60	R = 0.60	R = 0.60

Rep – Total number of neonates.

Conc – Concentration of NaCl.

The clonal lineage N91 was the one exhibiting the highest tolerance when considering the somatic growth rate. Only the two highest concentrations decreased the growth of N91 females (<10% growth inhibition; F_5,53_ = 6.79, p = 10^−5^, Dunnett's test: p<0.01). Conversely, clonal lineage N116 exhibited the highest sensitivity to NaCl when considering this parameter, with a concentration of 0.72 g/L of NaCl significantly reducing its somatic growth rate (7%) (F_5,50_ = 25.2, p = 10^−3^; Dunnett's test: p<0.003). Finally, all tested concentrations of NaCl provoked a significant decrease in the intrinsic rate of natural increase of all clonal lineages, except for E89. For this clonal lineage, only the three highest concentrations of NaCl caused a significant reduction in the intrinsic rate of natural increase (F_5,54_ = 854, p = 10^−2^; Dunnett's test: p<10^−4^).

No correlation was found between LC_50_ and EC values of NaCl (Pearson's correlations for EC_20_: r≤|0.51|, p≥0.38).

### Association between resistance to Cu and to NaCl

Despite the above mentioned similar sensitivity of clonal lineages to lethal levels of NaCl (Table 2), its association with the sensitivity to lethal levels of copper was tested. Significant correlations were not found after 48-h exposures (Pearson's correlation: r = |0.012|, p = 0.98). A negative associations between the resistance to lethal levels of copper (LC_50,48 h_) and to sublethal levels of NaCl causing impairment on reproduction was found (Pearson's correlations for EC_25_ and EC_50_: r = 0.90, p = 0.04 and r = 0.63, p = 0.253, respectively). Clonal lineages E99 and E89 and clonal lineages E99, E89 and N116, were, respectively, inversely sensitive to lethal and sublethal levels of copper and NaCl (Table 2). The clonal lineage N31 was found to be co-critically sensitive to lethal levels of copper and NaCl (Table 2).

Finally, within genotypes variation, measured as the mean of relative spreads of resistance (observed range inside brackets), was 23 (5 to 52%), 15% (6 to 21%), and 70% (42 to 120%) for copper, lethal levels of NaCl, and sublethal levels of NaCl, respectively; with two out of five clonal lineages being critically sensitive to each of these metals.

## Discussion

The range of lethal and sublethal sensitivities of the five clonal lineages of *D. longispina*, obtained in the present work, were similar to those reported in the literature for other clonal lineages of this species and also for other cladoceran species. Gonçalves et al. [34] reported for another clonal lineage of *D. longispina*, an LC_50,48 h_ of 2.9 g/L and an EC_50_ for reproduction of 2.2 g/L of NaCl. These values are equal to those computed in the present study for the clonal lineages N116 and E89, respectively. *Daphnia longispina* (LC_50,48 h_ = 2.69 g/L) was found to be slightly more resistant to lethal levels of NaCl than *Ceriodaphnia dubia* (1.59 g/L; [34]) and than *D. ambigua* (2.00 g/L; [34] Harmon et al., 2003), being more sensitive than *D. magna* (5.48, 5.5 and 5.9 g/L; [3], [8], [34] respectively). The tolerance of the five clonal lineages to sublethal levels of NaCl (EC_50_ for reproduction from 0.99 to 2.15 g/L) was also within the range reported in the literature for other cladocerans, namely *C. dubia* (1.35 g/L; [35]), *D. ambigua* (0.65 g/L; [35]) and *D. magna*, (5.0 g/L; [34]). It is also worth of notice that the intervals registered between concentrations inducing lethal and sublethal effects in *D. longispina* were very narrow (LC_50,48 h_∶EC_50_ ratios from 1.3 to 2.5). This result is in line with other works addressing the effects of NaCl to other cladocerans, namely *C. dubia* (1.2) and *D. ambigua* (3.1) [35].

No correlations between lethal and sublethal responses to NaCl were observed, supporting the endpoints (dis)association hypothesis [15]. This was an expected result, because the mechanisms involved in responses to lethal and sub lethal levels of contamination are probably different. The former are likely to involve one or a few specific mechanisms, such as metallothionein production and changes in enzymatic activities, while the latter are likely to involve more than one general physiological mechanism [36], [37]. Such a lack of association is in line with other works carried out with clonal lineages of the cladocerans *D. longispina* and of *C. pulchella* [25], [38], [39]. However, the occurrence of a positive association between lethal and sublethal responses could occur [14], [15]. For example, a positive correlation between lethal and sublethal (avoidance behaviour) sensitivity to copper in twelve lineages of *D. longispina* was found by Lopes et al. [40]. Furthermore, genetic processes like pleiotropy and epistasis may also lead to an association between different levels of responses and different chemicals [15], [41].

In the published literature, no work was found addressing a possible association between the tolerance to copper and to sodium chloride. However, some works reporting the existence of co-tolerance between NaCl and other cations, or Cu and other metals have been published (e.g. [20], [21], [38], [41], [42], [43]). As an example, Langdon et al. [43] showed that the earthworm *Lumbricus rubellus* inhabiting arsenic–contaminated soils developed tolerance to this metal. In follow-up studies, Langdon et al. [44] showed that this population of earthworms also exhibited increased tolerance to copper. Lopes et al. [38], also detected a positive relationship in genetically determined responses of clonal lineages of *D. longispina* to lethal levels of copper and zinc.

In the present study, an association between responses to lethal levels of Cu and NaCl was not observed, suggesting that different mechanisms are involved in lethal tolerance to each of the chemicals. In fact, though copper homeostasis is related, among others, with osmoregulatory mechanisms (e.g. active transport of copper may also occur through Na^+^/K^+^ ATPases), which are also related with the passive or active transport of Na^+^ and Cl^−^ ions, it is expected that, for example, metallothioneins will be also involved in the tolerance of clonal lineages to copper [45], [46]. Actually, a few studies already reported the implication of metallothioneins in the detoxification of Cu in crustaceans [45], [47]. Although the lack of association at lethal levels of both chemicals, a negative association was found between responses to lethal levels of Cu and to sublethal levels of NaCl (reproduction). Though, information is needed to understand at the physiological level such negative association, it can be hypothesized that this may be somehow related with the fact that in neonates and adults of daphnids Na^+^ transport mechanisms may have a different importance in the transport of this metal. For example, Bianchini and Wood [48] reported that uptake mechanisms in neonates of *D. magna* have a lower affinity for Na^+^, thus, favoring the ionoregulatory metals, such as copper, which could lead to a higher accumulation of metals in neonates (the only life cycle stage exposed to lethal levels of NaCl and Cu in the present study). These authors also suggested that a major mechanism controlling Na^+^ transportation in neonates of *D. magna* involves an epithelial Na^+^ channel associated with a V-H^+^-ATPase, which could also favor the accumulation of metals in neonates [48]. This higher accumulation of metals could involve a higher production of metallothioneins to bind the metals and capture them in lysosomes to avoid adverse effects inside the cells. However, these mechanisms are not as active in adults as in neonates, being others mechanisms more relevant in the transport of Na^+^, which may also influence the homeostasis of copper. These changes in osmoregulatory mechanisms may be related with the negative association between Cu and NaCl, as adults were exposed to the sublethal levels of NaCl.

## Conclusions

The negative association between lethal levels of Cu and sublethal levels of NaCl, as well as the inverse sensitivity observed in clonal lineages E99, E89 and N116 for NaCl an copper, supports the multiple stressors differential tolerance hypothesis, which predicts a genetic erosion augmentation at sequential pulses of different contaminants, and, therefore, may have implications for ecological risk assessment. If a population genetically eroded by past partially lethal copper contamination becomes exposed to saltwater, then it would be at a higher risk of a further loss of genetic diversity and, eventually, to extinction than non-eroded populations. As mentioned in the introduction, within such a scenario of a negative linkage between the tolerance to the two contaminants, at least some of the genotypes remaining in the population (those tolerant to Cu) may disappear after the exposure to NaCl to which they are sensitive to. As a gradual salinization of coastal freshwater lagoons is expected, the negative association between resistance to lethal levels of Cu and to low sublethal levels of NaCl (especially with the EC_25_) is likely to substantially increase the risk of extinction of genetically eroded populations due to a past copper exposure. This also holds true for the reverse sequence of exposures with partially lethal copper inputs during or after salinization.
